# An evaluation of machine learning classifiers for next-generation, continuous-ethogram smart trackers

**DOI:** 10.1186/s40462-021-00245-x

**Published:** 2021-03-30

**Authors:** Hui Yu, Jian Deng, Ran Nathan, Max Kröschel, Sasha Pekarsky, Guozheng Li, Marcel Klaassen

**Affiliations:** 1 Centre for Integrative Ecology, School of Life and Environmental Sciences, Deakin University, Geelong, Victoria Australia; 2 Druid Technology Co., Ltd, Chengdu, Sichuan China; 3 The Movement Ecology Laboratory, Department of Evolution, Systematics, and Ecology, Alexander Silberman Institute of Life Sciences, The Hebrew University of Jerusalem, Jerusalem, Israel; 4 Department of Wildlife Ecology, Forest Research Institute of Baden-Württemberg, Freiburg, Germany; 5 Chair of Wildlife Ecology and Wildlife Management, University of Freiburg, 79106 Freiburg, Germany; 6 Northwest Institute of Eco-Environment and Resources, Chinese Academy of Sciences, Lanzhou, Gansu China

**Keywords:** Accelerometer, Behaviour classification, On-board processing, ANN, Random forest, XGBoost

## Abstract

**Background:**

Our understanding of movement patterns and behaviours of wildlife has advanced greatly through the use of improved tracking technologies, including application of accelerometry (ACC) across a wide range of taxa. However, most ACC studies either use intermittent sampling that hinders continuity or continuous data logging relying on tracker retrieval for data downloading which is not applicable for long term study. To allow long-term, fine-scale behavioural research, we evaluated a range of machine learning methods for their suitability for continuous on-board classification of ACC data into behaviour categories prior to data transmission.

**Methods:**

We tested six supervised machine learning methods, including linear discriminant analysis (LDA), decision tree (DT), support vector machine (SVM), artificial neural network (ANN), random forest (RF) and extreme gradient boosting (XGBoost) to classify behaviour using ACC data from three bird species (white stork *Ciconia ciconia*, griffon vulture *Gyps fulvus* and common crane *Grus grus*) and two mammals (dairy cow *Bos taurus* and roe deer *Capreolus capreolus*).

**Results:**

Using a range of quality criteria, SVM, ANN, RF and XGBoost performed well in determining behaviour from ACC data and their good performance appeared little affected when greatly reducing the number of input features for model training. On-board runtime and storage-requirement tests showed that notably ANN, RF and XGBoost would make suitable on-board classifiers.

**Conclusions:**

Our identification of using feature reduction in combination with ANN, RF and XGBoost as suitable methods for on-board behavioural classification of continuous ACC data has considerable potential to benefit movement ecology and behavioural research, wildlife conservation and livestock husbandry.

**Supplementary Information:**

The online version contains supplementary material available at 10.1186/s40462-021-00245-x.

## Background

Biologging not only advances research in movement ecology, behavioural ecology and applied ecology, but also continues to contribute increasingly to wildlife conservation and livestock management [1]. In addition to the position of tracked animals in time, advanced biologging technologies also provide opportunities for additional environmental data collection such as ambient temperature, light intensity and water depth, and data related to logger carriers such as heart rate, energy expenditure and behaviour [2–4]. Moreover, the shrinking size and increasing energy efficiency of current trackers progressively enables studies across a wide range of animal taxa in a great variety of environments [5, 6].

Among add-on sensors in advanced biologging, accelerometers have gained popularity in the recent three decades [7]. An accelerometer is an electromechanical device measuring acceleration, most commonly along all three dimensions (i.e. triaxial accelerometry). When attached on animals, accelerometry (hereafter ACC) reflects two aspects of movement: static acceleration and dynamic acceleration. Static acceleration is due to gravitational force acting on the accelerometer, which could be used to derive animal body posture [8]. Dynamic acceleration is due to changes of velocity caused by animal movement [8]. Based on these characteristics, at least four types of studies have been routinely conducted using ACC. Firstly, under the assumption that metabolic rate is positively correlated with the dynamic movement component, ACC data have been used to calculate overall dynamic body acceleration (ODBA) or vector dynamic body acceleration (VeDBA) as a proxy of an animal’s energy expenditure (e.g. [9, 10]). Secondly, for the interval between position fixes, which could in particular be of notable length in diving animals (e.g. [11]), body pitch (rotation around the lateral axis) and roll (rotation around the longitudinal axis) [12] derived from ACC data have been used in reconstructing the movement path of animals in between position fixes (e.g. [13]). Thirdly, an animal’s acceleration changes with pattern and frequency of locomotion, and also the environment in which it moves [14], thus allowing for the estimation of e.g. fin, wingbeat or stride frequency. ACC data have thus also allowed for biomechanics studies (e.g. [15, 16]). Fourthly, because animal behaviours consist of different postures and dynamic movement traits, ACC data has been used to classify animal behaviours (e.g. [7, 17–19]).

Quantifying animal behaviours by ACC requires elaborate processing to classify behaviour from raw sensor data [20]. In general, there are three approaches for behaviour classification from ACC data. The first of these is *direct classification* based on expert opinion. This approach may be suitable for behaviours that are characterized by easily detectable ACC signatures (e.g. [21]). However, lacking “ground-truthing” observations makes this arbitrary approach impossible to validate (excluding situations where validation is not required, such as discriminating active versus inactive behaviour). In addition, an expert’s judgements make this approach difficult to generalize across studies involving different researchers. Secondly, an approach using *unsupervised machine learning* or clustering can be used, which groups ACC data based on commonalities in the ACC signal, where the grouping need not necessarily be associated with different behaviours. An example of such technique is k-means clustering (e.g. [17, 22]). Finally, *supervised machine learning* classification approaches based on “ground-truthing” observations can be used, in which behaviours are assigned to ACC data for model training. Using this approach researchers label captive or free-ranging animals’ ACC data with a specified set of behaviour categories through direct observation or video-taping (e.g. [23, 24]). ACC data are commonly recorded in fixed (but possibly user-adjustable) length segments called “bouts”, sometimes also called “bursts” or “epochs”. Bout length is selected by the user according to the study goals, and is often set to contain only a single episode of the behaviour(s) of interest. Then, for each bout, the ACC data are used to calculate a range of mathematical features such as mean, standard deviation, correlation coefficient between the ACC axes, etc. [7]. In the next step, the supervised machine learning method is trained to use these feature data in automatically classifying the ACC data into appropriate behaviour categories. Commonly applied supervised classification methods for animal behaviour classification include linear discriminant analysis, support vector machine, decision tree, random forest, and artificial neural network [19]. Generally, the trained classifiers are validated with a validation set of labelled ACC data.

In current tracking research of free-ranging animals, data recorded by trackers is either stored on board (e.g. [22]) or transmitted through mobile networks (e.g. [25]) or satellites (e.g. [26]). The amount of data that can be logged and transmitted from a tracker is often constrained by battery capacity, or solar radiation if solar-powered trackers are being used. Compared with data logging, the transmission process in trackers particularly consumes much battery power [27]. Thus, data volume through transmission and transmission rate are often limited by power supply. In addition, the amount of data that can be transmitted via satellites is limited [28]. Given these two limitations, many studies have applied intermittent sampling of ACC data rather than continuous recording (e.g. [29]). Obviously, intermittent sampling comes at the expense of information. On-board storage of continuously sampled ACC data is an alternative, but increases power consumption and requires large device storage, which might increase the device’s weight and size. Moreover, it often requires recapture or tag retrieval (e.g. by capturing the animal or tag automatic drop-off) for data downloading. Thus, studies using continuous ACC data sampling either recaptured their study objects multiple times to download data (e.g. [30]) or only tracked animals for a short period of time (e.g. [31]). Multiple recaptures may not only bear a time and financial cost but also influence the behaviour of animals, while studies tracking animals for a short period of time may have less ecological significance.

On-board data processing may provide a solution to data-storage and data-transmission limitations and may extend the possibilities to continuously record the behaviour of tracked animals for prolonged durations of time. One way of on-board data processing is to transform raw ACC data into features for transmission or downloading (e.g. [32]). Alternatively, the complete classification procedure can be implemented in the tag that only provides a data stream of classified behaviours instead of the raw data. There are only a few on-board behaviour classification studies currently, and this is mainly due to the large amount of data involved and the complexity of the processing procedures [28]. Roux et al. [33] used custom-made loggers for on-board behaviour classification of *Dohne Merino* sheep, with five behaviour categories, and rhinoceros (*Ceratotherium simum* and *Diceros bicornis*), with three behaviour categories. They used linear discriminant analysis on an initial ACC test data set, after which the logger was programmed to use the resulting algorithm for subsequent recording of behaviours via ACC. In another on-board data processing study in juvenile southern elephant seals (*Mirounga leonine*), Cox et al. [28] identified foraging behaviour by user-defined thresholds based on expert opinion and validated their method by comparison of on-board calculated foraging behaviours with foraging behaviours identified from archived raw ACC data. Korpela et al. [34] used on-board behaviour classification through ACC data to detect foraging behaviour of seabirds, triggering video-loggers to record the foraging behaviour. Moreover, to our knowledge, no published behaviour classification has compared the practicability of the aforementioned more sophisticated classification methods (i.e. support vector machine, decision tree, random forest, and artificial neural network) on-board, probably due to limitations of tracker storage and battery capacity. For these behaviour classification methods to be successful, feature calculation and selection are crucial elements [35]. Computing and using large numbers of (complex) features in on-board behaviour classification would require abundant storage and be energy consuming. Thus, developing ways to reduce computation while maintaining high behaviour classification accuracy also requires consideration.

In this study, we tested six supervised machine learning methods. Five methods among the six were applied in other studies, including linear discriminant analysis (LDA) (e.g. [33]), decision tree (DT) (e.g. [24]), support vector machine (SVM) (e.g. [36]), random forest (RF) (e.g. [37]), and artificial neural network (ANN) (e.g. [19]). We added extreme gradient boosting (XGBoost) to our study given its good performance in Kaggle machine learning competitions [38]. XGBoost is a tree-based model which carries out the gradient boosting tree algorithm with high speed [38]. In order to further reduce on-board calculation and thus power demand, we also investigated these models’ performance using greatly reduced feature sets, aiming at minimizing storage requirements and runtime while maintaining high classification accuracy. We applied our proposed animal behaviour classification from ACC data to different animal taxa and different tracker-attachment methods (i.e. ear tags, backpacks, neck collars, and leg bands) to broaden the scope of our analysis. The combination of continuous behaviour monitoring and GPS locations of tracked animals will provide researchers with a powerful tool to conduct research within the movement ecology realm [39] and we therefore hope our study will facilitate the development of next generation “smart” trackers that have these features.

## Methods

### Data sources

Five different sets of ACC data were used including unpublished data from two Chinese Holstein dairy cows (*Bos taurus*) and two common cranes (*Grus grus*), and published data collected on eight roe deer (*Capreolus capreolus*) [40], 32 griffon vultures (*Gyps fulvus*) [19] and 23 white storks (*Ciconia ciconia*) (data available in AcceleRater website: http://accapp.move-ecol-minerva.huji.ac.il/, see [41]). Ear-mounted loggers in ruminants are particularly suitable to pick up foraging and ruminating, where sudden change of daily rumination time is a potential indicator of oestrus or illness [42]. The two lactating dairy cows were held in pens measuring 15 m × 8 m and were fitted with accelerometer data loggers (18 g test model from Druid Technology Co., Ltd., China) in Chengdu, China, between 2017/12/29 and 2018/01/26. The ACC data logger was programmed to record at 25 Hz with 12-bit resolution in a ± 4 g (i.e. 1 g = 9.8 m/푠^2^) range. The loggers were glued on the already present Radio Frequency Identification Device (RFID) ear tags of each dairy cow. The triaxial ACC data was continuously recorded and transmitted through Bluetooth 4.0 to an Android cell phone. Behavioural data was collected simultaneously through direct visual observation by Hui Yu using a specially designed cell phone application “Utopia Druid”. In total, 12.4 h of labelled ACC data across both dairy cows were collected. Cow ACC data was labelled using three behavioural categories: eating (i.e. ingesting food), ruminating (i.e. rechewing the cud to further help break down the earlier ingested plant matter), and other (i.e. behaviours not labelled as eating and ruminating).

Two captive common cranes, one adult in a pen and one juvenile in a large semi-natural area with trees and a bog, were fitted with GPS-ACC transmitters (OrniTrack-L40, Ornitela, Vilnius, Lithuania) on a leg band. The ACC data was recorded for 3.8 s at 10.54 HZ in 3-axes, every 30 s. The birds were videorecorded allowing manual matching with the recorded ACC data. In total 1830 of these 3.8 s long ACC observation bursts (i.e. ~ 15 h of observation) were thus labeled using four behavioural categories: feeding (i.e. ingesting food or collecting food without movement), foraging (i.e. moving with head down while looking for food and occasional swallowing of food), moving (i.e. walking or running) and resting (i.e. standing or preening).

Eight roe deer were tracked with GPS-ACC collars (e-obs GmbH, Munich, Germany). The ACC data was recorded for 9.1 s at 10.54 Hz at either 1 min or 15 s intervals. In total 6158 ACC observation bursts were labelled totalling ~ 30 h of field observation. Thirty-two griffon vultures were tracked with GPS-ACC backpacks (e-obs GmbH). ACC data was recorded for either 9.1, 16.2, 20.4, or 24.6 s at 3.3 Hz at 10 min intervals. In total 488 ACC observation bursts were labelled totalling ~ 80 h of field observation. Twenty-three white storks were tracked with GPS-ACC backpacks (e-obs GmbH). The ACC data was recorded for 3.8 s at 10.54 Hz at 5 min intervals. In total 1746 ACC observation bursts were labelled during ~ 145 h of field observation.

For the published studies we combined a number of behaviour categories for a variety of reasons. For roe deer [40] we combined “galloping” and “trotting” into “running” to create sufficient samples for cross validation. For the same study we also combined “lying” and “standing” behaviours into “static” since the ACC tracking neck collars used in their study did not allow for discrimination between these two static postures. The roe deer dataset thus comprised five behaviours including browsing, running, static, walking and other (i.e. shaking, scratching with antler, scratching with hoof, grooming). For griffon vulture [19] we dropped the “lying down” behaviour because its sample size was too small for cross validation and the behaviour was also not suitable to be combined with any other behaviour classes. The griffon vulture dataset thus ultimately had five behaviours: active behaviour (preening, running and other active behaviours on the ground), active flight (flapping), passive flight (soaring-gliding), eating and standing. For white stork [41] we kept all original five behaviour categories, which included: active flight, passive flight, sitting, standing and walking.

### Segmentation and feature calculation

Each of the five ACC datasets was divided into bouts where the bout length was chosen such as to have a maximum of ACC information while still reflecting only one specific behaviour type. Any bouts reflecting more than one behaviour were pruned from the datasets. For the dairy cow dataset, the bout length was set to 1 min (i.e. 1500 ACC records, where 741 out of 745 contained one behaviour type only and were retained for training and validation). This relatively long bout duration was chosen because dairy cows typically show one type of behaviour for prolonged periods of time and do not change behaviour frequently [36]. Bout lengths of common crane, griffon vulture, roe deer and white storks were considerably shorter. For common crane, the original ACC burst lengths of 3.8 s were used as bouts (i.e. 40 ACC records, where 1385 out of 1830 bouts retained). Also, for white stork the burst length of 3.8 s was used as a bout (i.e. 40 ACC records, all 1746 bouts retained). In the griffon vulture study, bout lengths varying between 9.1 and 24.8 s were originally used [19], which we altered to a standard 9.1 s (30 ACC records, where all thus resulting 815 bouts were retained). For roe deer (96 ACC records), the original bouts contained up to five behaviours. We therefore halved the bout length to 48 ACC records (i.e. 4.6 s) and retained bouts with only one specific behaviour (10,576 out of the resulting 12,316 bouts were retained).

For *full feature set* calculation, we used a total of 78 different features (also called *summary statistics*; Table 1) being calculated for each bout. However, among others due to correlation between features (see Machine-learning algorithms, below), the number of features could potentially be greatly reduced without marked reduction in explanatory power. We thus also used a greatly *simplified feature set*, consisting out of four or five features depending on tracker placement. In white stork and griffon vulture (backpack trackers in line with the thoracic spine), roe deer (neck collars in line with the cervical spine) and common cranes (leg mounted trackers in line with the tibia), the surge (motion along the longitudinal axis) and heave (motion along the vertical axis) axes were considered the two main axes related with body movement, of which we took both the mean and standard deviation of each in addition to ODBA (i.e. five features in total). Of these five features, the means of the surge and heave axes have earlier been shown to capture body posture information [35]. We consequently used their standard deviations to capture dynamic movement. We also included ODBA in all simplified feature sets as it captures dynamic movement strength and has been successfully used as an index of energy expenditure [9]. For dairy cow (ear tags) we used only four features consisting of the mean and standard deviation of the heave axes, ODBA and the main frequency component of the heave axis. The latter was included aiming at recording jaw movements. Despite its successful use in other studies (e.g. [17]), we abstained from using frequency information for any of the others species for two reasons: firstly, sampling frequency may not always be adequate to log useful frequency information [19] and secondly, frequency information requires computationally demanding Fourier transformation, whereas we were aiming to reduce computational demands as much as possible.

**Table 1:** Description of 78 features used in the behavioural classifications of triaxial accelerometer data

Feature	Explanation
Mean	Mean of measurement along each axis
Variance	variance of measurement along each axis
Standard deviation	Standard deviation of measurement along each axis
Coefficient of variance	Coefficient of variance of measurement along each axis
Skewness	Skewness of measurement along each axis
Kurtosis	Kurtosis of measurement along each axis
Maximum	Maximum value of measurement along each axis
Minimum	Minimum value of measurement along each axis
Range	Range of measurement along each axis
Euclidean norm	Euclidean norm of measurement along each axis
Covariance	Covariance of measurements between two axes
Correlation	Pearson correlation of measurements between two axes
Mean difference	Mean difference of measurements between two axes
Standard deviation of difference	Standard deviation of measurements between two axes
Variance of static body acceleration	variance of static body acceleration along each axis
Variance of dynamic body acceleration	variance of dynamic body acceleration along each axis
Mean dynamic body acceleration	Overall dynamic body acceleration along each axis
Maximum dynamic body acceleration	Maximum value of dynamic body acceleration along each axis
Overall Dynamic Body Acceleration	Overall Dynamic Body Acceleration
Pitch	Pitch angle of the device
Roll	Roll angle of the device
Mean difference of continuous points	Mean difference between two continuous points along each axis
Variance of difference of continuous points	variance of difference between two continous points along each axis
Main frequency	Frequency at the main frequency along each axis
Amplitude of main frequency	Maximum amplitude of fft along each axis
25% quartile	25% quartile of measurement along each axis
50% quartile	50% quartile of measurement along each axis
75% quartile	75% quartile of measurement along each axis

### Machine-learning algorithms

All analyses were conducted in R [43]. LDA typically suffers from correlation of features [44]. To account for this, we deleted highly correlated features from the set of 78 features by setting the “cut off” parameter at 0.7 in the “findCorrelation” function in R package “caret”. We also applied DT (R package “rpart”), SVM with both a linear and a radial kernel (R package “e1071”), RF (R package “randomForest”), ANN (R package “nnet”), and XGBoost (R package “xgboost”). In order to achieve highest accuracies, we tuned parameter “cp” for DT (by function “train” in “caret” package), “gamma” and “cost” for SVM (by function “tune.svm” in “e1071” package), “mtry” and “ntree” for RF (“train” in “caret”), and “size” and “decay” for ANN (“tune.nnet” in “e1071”) (all parameters listed in Table S1). Performance of XGBoost showed little or no improvement by parameter tuning in all five datasets and its default settings (with “nrounds = 10”) were therefore retained. SVM with linear kernel proved to be inferior to SVM with radial kernel and only the latter was therefore retained.

### Training and validation of machine-learning algorithms

We conducted stratified 10-fold cross-validations for which each of the five ACC datasets was semi-randomly partitioned into ten subsets in which the various behaviour categories were proportionally equally represented as in the full dataset. For each of the classification models we conducted a training and validation procedure consisting of ten runs, where in each run another subsample was selected for validation and the remaining nine subsamples were used for training of the model. After each of the ten runs, we calculated a set of model evaluation metrics. In each iteration of the 10-fold cross-validations, the validation data was not used in the model training and acted as a test dataset exclusively. After all ten runs, the means and 95% confidence intervals of the evaluation metrics were calculated. For each behaviour category, we evaluated the prediction accuracy as an F1 score:

1 $$ \mathrm{F}1=\frac{2\ast Recall\ast Precision}{Recall+ Precision} $$

where $\mathrm{Recall}=\frac{TP}{TP+ FN}$, $\mathrm{Precision}=\frac{TP}{TP+ FP}$, *TP* is true positive, *TN* is true negative, *FP* is false positive and *FN* is false negative (see [41]).

Next, for each dataset, an overall accuracy score was calculated across all behaviours dividing the number of ACC data bouts where the behaviour was correctly classified by the total number of ACC data bouts (i.e. sum of correct and incorrect classifications):

2 $$ \mathrm{Overall}\ \mathrm{accuracy}=\frac{TP+ TN}{TP+ TN+ FP+ FN} $$

We further tested model performance using the simplified feature sets. We tuned parameters of DT, SVM and ANN. We set “ntree = 20” for RF and “nrounds = 5” for XGBoost to reduce model size. We conducted stratified 10-fold cross-validations for five datasets by the six models. We evaluated the model performances with F1 score and overall accuracy.

### Runtime of feature calculations

To evaluate the runtime of the different feature calculations, we programmed all functions for feature calculation on-board of a tracker with nRF52840 SoC (system on a chip), which has a 64 MHz microprocessor, 1 MB Flash memory and 256 KB RAM memory. The pseudocodes for these feature calculations are provided in Table S2. Since feature calculations for different datasets would follow the same procedures, we only used the white stork dataset as the demo dataset. The raw ACC data of the first bout of this demo dataset was pre-loaded together with the code for feature calculations on-board the tracker. Because all bouts in the dataset have the same length, the runtimes for the various features of this first bout were taken to be representative.

### Runtime and storage requirements of machine-learning classifiers

To evaluate the runtime of the different machine learning classifiers (i.e. the outcomes of the machine learning algorithms allowing behavioural prediction from ACC data), we programmed the classifier functions on-board the nRF52840 SoC described above. The classifier data included “SV”, “coefs”, “x.scale”, “rho” and “nSV” for SVM, “nconn”, “conn” and “wts” for ANN, trees for RF (using “getTree” in “randomForest” package), and trees for XGBoost (“xgb.dump” in “xgboost”). We tested classifiers with full and simplified feature sets. Only for the full-feature set RF model we set “ntree” to 200 instead of 800 since there was not enough on-board storage for 800 trees. Parameters for SVM, ANN and XGBoost and all other parameters except “ntree” for RF, were the same as listed in Table S1. The pseudocodes for these classifiers are provided in the supplementary material as Supplementary Algorithms 1, 2, 3 and 4. Aside from the classifiers, we also loaded the already calculated simplified feature set for the 1746 bouts in the white stork dataset. Because runtime may vary across bouts when using RF and XGBoost, we calculated the mean runtime across all 1746 bouts for each of the classifiers. The on-board storage requirements of the classifiers were also recorded.

## Results

**Fig. 1:**
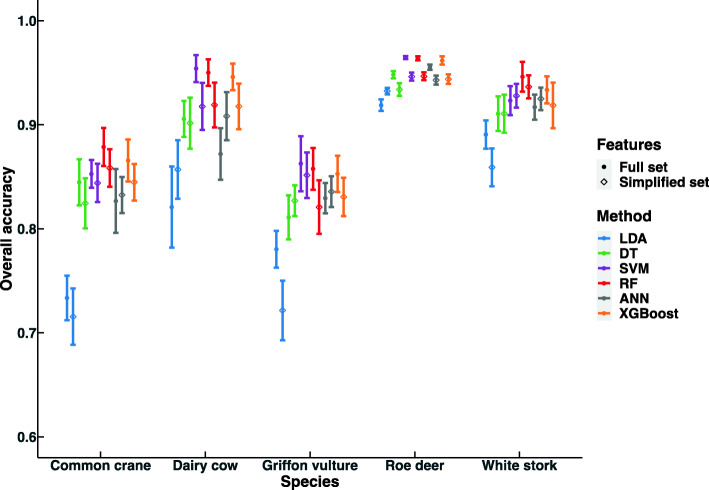
Comparison of overall accuracies of six machine learning methods across five different datasets encompassing Common crane, Dairy cow, Griffon vulture, Roe deer and White stork, with full features sets and simplified feature sets. Mean and 95% confidence interval using 10-fold cross-validation are presented. LDA: linear discriminant analysis, DT: decision tree, SVM: support vector machine, RF: random forest, ANN: artificial neural network, XGBoost: extreme gradient boosting

Using the full feature set, SVM, RF and XGBoost had indistinguishable performance (i.e. overlapping 95% confidence intervals) and always ranked as the top three models by overall accuracy across all five datasets (Fig. 1). DT and ANN performed better than LDA but worse than the top three models. Using the simplified feature set, DT, SVM, RF, ANN and XGBoost had similar overall accuracy across datasets except for the roe deer dataset, where DT had significantly lower overall accuracy than SVM and RF and also showed a tendency for a lower accuracy than ANN and XGBoost (Fig. 1). Five of the six models (i.e. DT, SVM, RF, ANN and XGBoost) generally had slightly lower accuracy when using a simplified compared to a full feature set, with a ~ 3.7% max mean accuracy difference. Interestingly, except in the roe deer case, using a simplified feature set ANN had higher overall accuracies than when using a full feature set, amounting to a maximum of 3.6% mean difference.

**Fig. 2:**
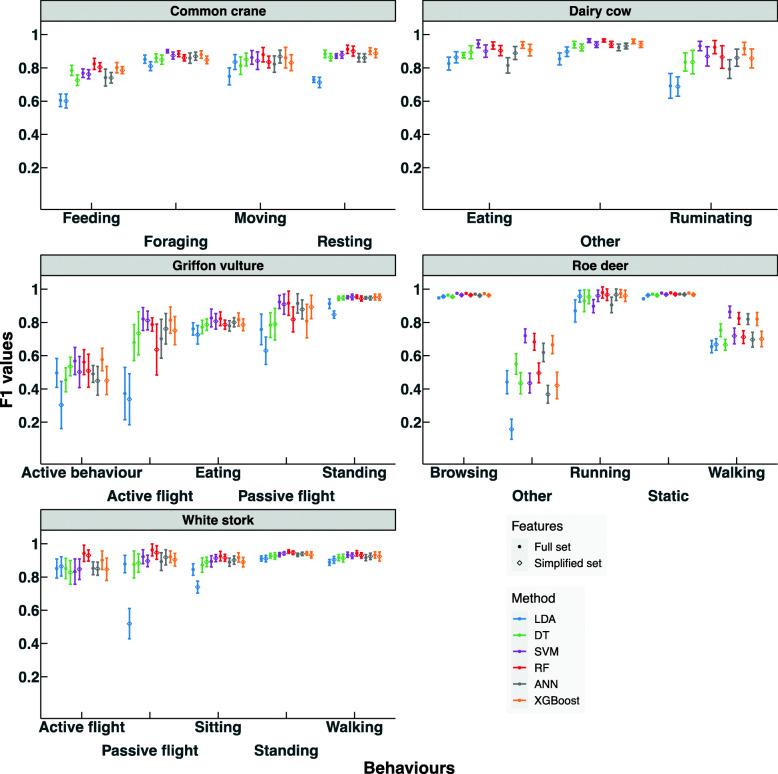
Comparison of F1 values of six different machine learning methods (see caption to Fig. 1 for abbreviations) across different behaviours in five datasets for Common crane, Dairy cow, Griffon vulture, Roe deer and White stork, with full feature sets and simplified feature sets. Mean and 95% confidence intervals using 10-fold cross-validation are presented

**Fig. 3:**
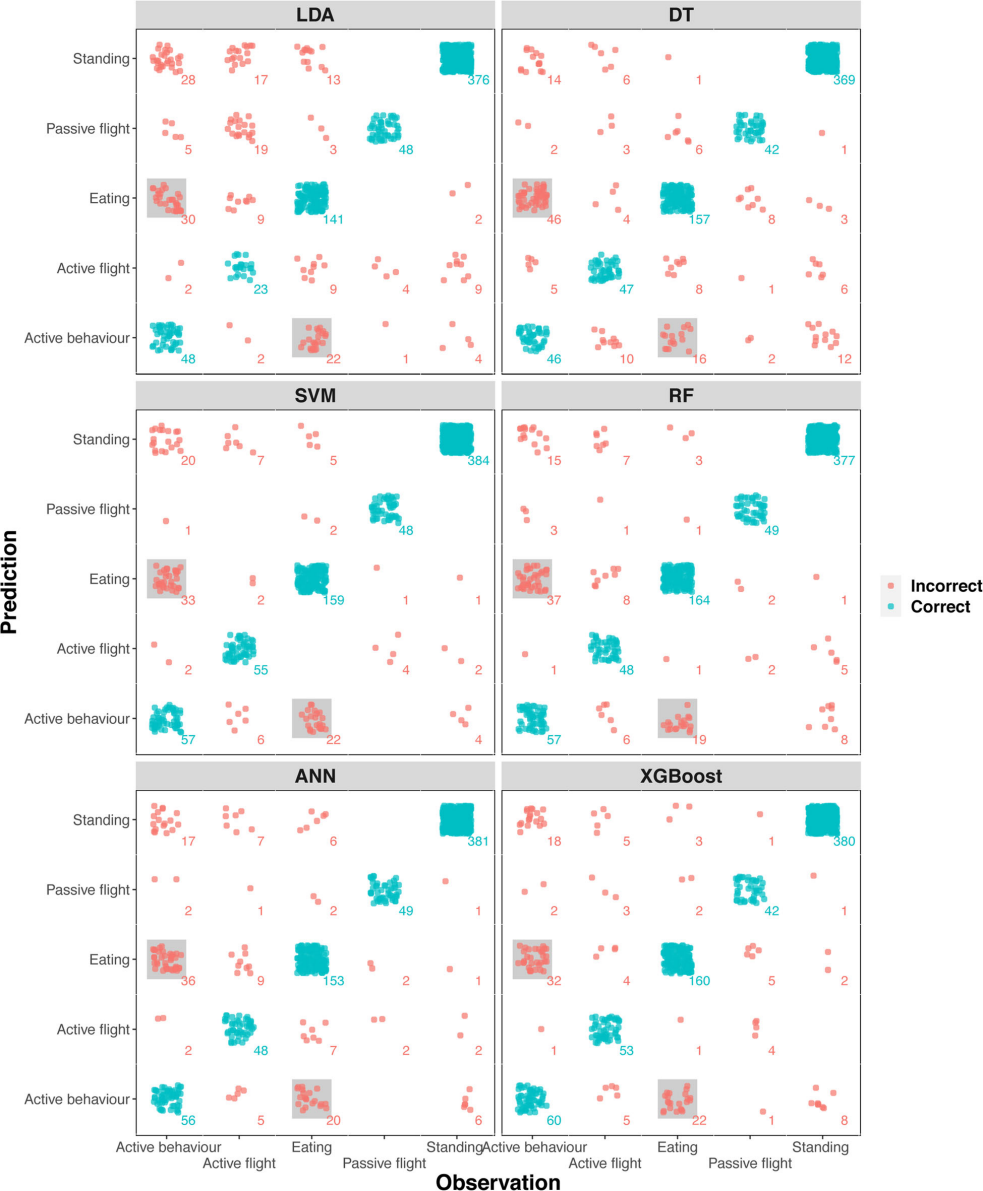
Confusion matrix plot of Griffon vulture dataset based on six machine learning models. Dots are coloured according to classification results (incorrect and correct; total sample size depicted for each behaviour combination) with grey shades highlight misclassifications between the behaviours “active behaviour” and “eating”

For each data set, the relatively low variation in the F1 scores of the different classification methods within a certain behaviour in comparison to the variation across the different behaviours was striking, either with full feature set or simplified feature set (Fig. 2). This suggests that, although some algorithms were clearly better than others, all machine learning methods had similar classification/mis-classification issues. This was best exemplified in the “active behaviour” and “eating” behaviours in griffon vulture with very low F1 values across all machine learning methods (Fig. 2), which was importantly due to misclassifications between the two behaviours (Fig. 3).

The on-board runtimes for the 78 features (Table 2) totalled 2.73 ms, whereas the runtime of the simplified feature set took only 0.31 ms or 11% of the time required for the calculation of the full feature set. Runtime evaluation of the four classifiers varied between 0.134 ms in XGBoost up to a whopping 34.628 ms in SVM with simplified feature set, and between 0.312 ms in XGBoost up to 43.042 ms in SVM with full feature set. While ANN had the lowest storage requirements of 3.42 kB with simplified feature set and 10.764 kB with full feature set, again SVM topping the charts with 26.724 kB with simplified feature set and 185.684 kB with full feature set (Table 3).

**Table 2:** On-board runtimes during feature calculations. Where features have been grouped in one row the total runtime for the calculations of all features is total listed. Under “Note” any dependencies for the calculation of the feature are listed. “Gross time” identifies the total runtime for the listed feature and its dependencies

Feature(abbreviation)	Net time(ms)	Gross time(ms)	Number of features calculated	Note
Mean(mean)	0.021	0.021	3	
Variance(var)	0.025	0.046	3	mean
Standard deviation(sd)	0.003	0.049	3	mean, var
Coefficient of variance	0.002	0.051	3	mean, sd
Skewness	0.361	0.41	3	mean, sd
Kurtosis	0.367	0.416	3	mean, sd
Maximum(max)	0.029	0.029	3	
Minimum(min)	0.029	0.029	3	
Range	0.001	0.059	3	max, min
Euclidean norm	0.017	0.017	3	
Covariance(cov)	0.033	0.054	3	mean
Correlation	0.002	0.084	3	sd, cov
Mean difference(meandiff)	0.026	0.026	3	
Standard deviation of difference	0.035	0.061	3	meandiff
Variance of static body acceleration Variance of dynamic body acceleration Mean dynamic body acceleration Maximum dynamic body acceleration Overall dynamic body acceleration	0.279	0.279	13	
Pitch	0.039	0.06	1	mean
Roll	0.049	0.07	1	mean
Mean difference of continuous points(meandl)	0.178	0.178	3	
Variance of difference of continuous points	0.025	0.203	3	meandl
Main frequency Amplitude of main frequency	0.258	0.258	6	
25% quantile 50% quantile 75% quantile	0.94	0.94	9

**Table 3:** On-board runtime and storage requirement of four machine learning methods with full feature sets and simplified feature set

	SVM	RF	ANN	XGBoost
**Runtime(ms)**				
Full feature set	43.042	2.154	1.044	0.312
Simplified feature set	34.628	0.186	0.826	0.134
**Storage requirement(kB)**				
Full feature set	185.684	164.808	10.764	24.3
Simplified feature set	26.724	23.064	3.42	13.164

*SVM* Support vector machine, *RF* Random forest, *ANN* Artificial neural network, *XGBoost* Extreme gradient boosting

## Discussion

In this study, we compared six machine learning methods in their suitability to predict behaviours using ACC datasets from five different species. Generally, the classification accuracy across all five datasets was better in SVM, RF, ANN and XGBoost than when using LDA and DT. Yet, using these models with full feature sets can be computationally demanding, potentially limiting their use for on-board behaviour classification. However, we next showed that calculation demand of the six models could be greatly reduced through simplified feature selection and by reducing the number of model parameters (i.e. “ntree” of RF and “nrounds” of XGBoost), without substantial reduction in accuracy. After comparing storage requirements and runtimes of the six models and given their similar prediction accuracy, ANN and XGBoost therewith have great potential to be general-duty, on-board classification methods for continuous behaviour tracking using ACC.

In our study, SVM, RF, ANN and XGBoost generally performed well in regard to F1 score and overall accuracy, either with full or with simplified feature sets. Also other studies found that the four methods – SVM, RF, ANN and XGBoost – generally had good performance on classification tasks. Weegman et al. [45] used the on-line animal behaviour classification tool [41] in a behavioural study of Greenland white-fronted goose (*Albifrons flavirostris*), with RF reportedly having the highest classification accuracy of various models tested. Resheff et al. [41] found that ANN performed better than 6 other algorithms examined for the vulture dataset although the RF method performed nearly as well (overall accuracy of 84.84% vs 84.02%, respectively). Rotics et al. [46] found that SVM performed best of all other methods tested on an extended white stork dataset (i.e. 3815 ground-truthed ACC bouts) from the one used in this study, reaching an overall accuracy of 92%. Yet, Sur et al. [37] found that k-nearest neighbour is better than RF to distinguish more detailed behaviours such as straight flights and banking flights in golden eagle (*Aquila chrysaetos*), although the two methods both achieved high accuracies in classifying basic behaviours including flapping flight, soaring flight and sitting. However, their conclusions may have been flawed since they trained and evaluated RF with features, whereas k-nearest neighbours was trained and evaluated with raw data.

XGBoost has never before been used in animal behaviour classification. However, Ladds et al. [31] combined RF and Gradient Boosting Machine learning to form a super learner for behaviour classification in three different species of fur seals (*Arctocephalus pusillus doriferus, Arctocephalus forsteri* and *Arctocephalus tropicalis*) and Australian sea lions (*Neophoca cinerea*). The super learner improved ~ 1.4% overall accuracy over RF alone. XGBoost is a scalable tree boosting method which proved to be better than other tree boosting methods and RF [38]. Thus, it’s not a surprise that XGBoost had good performance in this study.

Obviously, behaviour classification accuracy from ACC data not only relies on the algorithms of choice, but also the functioning and placement of the ACC device, the definition of the behaviour set and the segmentation of the ACC data. For instance, the classification problems distinguishing between active behaviour and eating of griffon vulture (Fig. 3) may have arisen from device placement. The griffon vultures were tracked by backpacks, ACC data being importantly influenced by trunk movements with possibly similar triaxial signal patterns between some activity behaviours and eating. In a study comparing behaviour classification performance for Canada goose (*Branta canadensis*) equipped with neckbands and backpacks, Kölzsch et al. [24] possibly unsurprisingly found that neckbands were better able to distinguish behaviours involving elaborate head movements whereas backpacks were better at behaviours related to body movement. Defining the behavioural set may also be crucial. Having a “remainder” behavioural category such as “active behaviour” in griffon vulture may be ecologically meaningful but may be problematic to differentiate from more specific behavioural categories. A few studies compared behaviour classification performance with varying numbers of behaviour categories, finding that fewer categories generally yield higher classification accuracy [20, 31]. Variations in ACC data belonging to the same behaviour type will also influence behaviour classification accuracy. Accelerometers that shift their position on tracked animals cause intra-individual variation. This source of variation is practically impossible to measure in most wild animals, hence difficult to assess. Intraspecific variation among tracked animals (e.g. age, sex and body mass) and differences in placement of the accelerometer may cause inter-individual variation. These sources of variation are commonly measured before an animal is tagged and released, hence their effects can, in principle, be assessed. For the white storks in this study, the classification accuracy appeared unaffected by inter-individual variation, neither wing length (*p* = 0.76, R^2^ = 0.005), weight (*p* = 0.45, R^2^ = 0.03), nor sex (*p* = 0.33, R^2^ = 0.05) having and effect on classification accuracy. Using ACC data collected from multiple individuals for model training may result in more robust classifiers [47]. Furthermore, minimising inter and intra-individual variation in behaviour-specific ACC signals variations as much as possible remains of paramount importance and can sometimes be achieved. For example, in the roe deer case, the weight and the low center of gravity of the batteries prevented the neck collar from turning around the neck and made sure that the accelerometer remained in a dorsal position. Also, a thorough description and consistency of tracker attachments [48] would help minimizing inter-individual variations. Finally, when committing to on-board behaviour classification researchers should consider validating their models over time when there is a possibility to do so.

The use of simplified feature sets for animal behaviour classification is not only valuable for on-board calculation, but potentially also important for broader use of ACC in animal behaviour studies. Generally, the performance of models with 78 features was only marginally better than that for models using simplified features sets, which was also found in [49]. The explanation for this finding importantly resides in the fact that the original 78 features contain highly correlated features that contain very little additional information [50]. Although the potential on-board calculation models – SVM, RF, ANN and XGBoost – can adequately cope with correlations in data sets, correlation among features unnecessarily consumes computational power and data storage. In addition, some features may have a negative effect on model performance, such as observed for the ANN model when used on the dairy cow, common crane, griffon vulture and white stork datasets.

Parameter tuning is crucial for the performance of machine learning models. In this study, we noticed that SVM and ANN need careful tuning to achieve good performance. Importantly, when the input features for model training were changed from full feature sets to simplified feature sets, the parameters of SVM and ANN needed retuning, requiring hours of computation time. In contrast, RF and XGBoost proved much more user friendly, performing well using most of their default settings for all datasets used, except for user defined “ntree” in RF and “nrounds” in XGBoost (see Table S1).

We segmented ACC data using fixed time intervals with the unavoidable risk of obtaining bouts containing multiple behaviour types, potentially limiting machine-learning classification accuracy. Indeed, Bom et al. [51] showed that variable instead of fixed time segmentation improved behaviour classification in crab plover (*Dromas ardeola*). Combination of variable time segmentation and ANN or XGBoost might thus be an interesting avenue to further improve behaviour classification accuracy and further reduce on-board computational demands. Even further improvements might be achieved by a combination of unsupervised and supervised machine learning methods. This relates to the common deficiency of supervised machine learning models to accurately classify rare behaviours. Such rare behaviors, however, might constitute the main focus of particular studies, and machine-learning methods could be selected according to their ability to identify particular behaviors (e.g. [52]), including rare ones. Whereas some rare behaviours may still be observed and recorded for model training, their sample size may be too small for adequate model training (e.g. [53]). This problem may be aggravated when behaviours of importance are only temporarily (e.g. seasonally) expressed, such as mating and incubation behaviours during the breeding season and animals moving through snow in winter. To overcome this problem, future studies could investigate ways to flexibly combine supervised and unsupervised machine learning to also enable the classification of behaviours in the absence of data for ground truthing. Another solution might be to retain those data that cannot be classified with high accuracy or transmit data summaries when such unclassifiable events occur [32]. This procedure would allow for more precise classification in the lab, whereas all other data are deleted. When the rare or seasonal behaviours are not the focal behaviours of key interest, a basic behavioural classification (as e.g. just stationary, foraging or transiting) might also be applied.

The simplified feature set had a greatly reduced on-board runtime from the full feature set. When the clock speed of the on-board microprocessor is settled, its energy consumption is proportional to runtime. Thus, the calculation of the here used simplified feature set only consumes ~ 11% of the energy needed to calculate the full feature set. As has become clear here, different trackers and animal systems may require alternative feature sets. Also, bout lengths and recording frequency may vary across studies. Thus, the absolute runtimes for feature calculation here presented for the white stork dataset (Table 2) are not directly transferable to other studies. Nevertheless, they may provide a very useful index for evaluating the relative runtimes and relative energy consumption requirements for a great variety of features and alternative feature sets in any study wishing to optimize real-time, on-board behaviour classification from ACC data.

With the simplified feature set, the on-board runtime test of classifiers showed that XGBoost was fastest, followed closely by RF. The XGBoost and RF classifiers make use of tree traversal (see Supplementary Algorithms 3 and 4), which only involves comparison operations. XGBoost was faster than RF in this test because it had fewer comparison operations involved. The ANN classifier mainly involves multiplications and additions (see Supplementary Algorithm 2). SVM took a much longer runtime than the other three classifiers. SVM involves kernel value calculations between the feature values of a behaviour bout and all the support vectors (see Supplementary Algorithm 1). The radial kernel SVM requires exponent operations, which take much longer time than add or multiply operations. Moreover, there were as many as 666 support vectors in the SVM classifier of the white stork example, explaining the contrastingly long runtime for this classifier.

With the simplified feature set, the on-board storage requirements of classifiers showed that ANN required least storage. The storage of the ANN classifier is related to the number of weights, whereas the storage requirements of the RF and XGBoost classifiers depends on the total number of nodes across all trees. The storage of the SVM classifier is related to the number of support vectors. Nevertheless, the maximum storage requirement among the four classifiers – 27 kB of SVM – is still very small considering the 1 MB Flash memory used here and the flash memory generally used in tracking devices.

The on-board runtime tests of feature calculation and classifiers showed that the development and operation of continuous-ethogram trackers is highly feasible from a power requirement perspective. Since the energy usage for ACC data recording is low [32], we here only take the energy usage for on-board feature calculation and behaviour classification into consideration. According to the here presented data, a 200mAh battery can support calculations of the fastest XGBoost classifier with five simplified features continuously for approximately 11,000 days (using the recording settings for white stork). Also, the most energy hungry SVM would be able to run for approximately 160 days. Whatever model used in this fashion, the data compression rate using this strategy is 240:1 (120 ACC records × 2 bytes versus 1 byte identifying behaviour type). In case data transmission is not feasible, this would enable as much as 46 days of behavioural data in 1 MB of memory (without timestamp). Finally, based on 3G-transmission estimates made in our lab in Chengdu, China, we estimated that transmission of 1 day of continuous raw ACC records would on average take 5862 s and consume 244.08mAh of battery power. By contrast, using the same network, transmission of 1 day of classified behaviour from ACC data would take only 52 s or less than 1% of the time, and consume 1.49mAh of battery power or only 0.6% of the energy needed for raw data transmission.

## Conclusions

On-board behaviour classification through ANN, RF or XGBoost may enable researchers to study wildlife behaviours at a detailed and continuous scale. This new tool therewith bears the promise of continuous and long-term behavioural studies, addressing a wide range of behavioural and ecological topics, including allowing precise, behaviour-triggered sampling (e.g. [34]) and interventions and experimental research. As an extension of such behavioural studies, the same data might also be used to assist in assessing energy expenditure, biomechanics and assist in track dead-reckoning. Other than wildlife ecology, continuous behaviour monitoring may also benefit captive and domestic animal management and welfare improvement.

## Supplementary Information

**Additional file 1: Supplementary Table 1.** Results of parameter tuning of four machine learning methods in five datasets (i.e., for Common crane, Dairy cow, Griffon vulture, Roe deer and Whites stork), with full feature sets and simplified feature sets. **Supplementary Table 2.** Pseudocodes for feature calculations used for on-board runtime evaluations. **Supplementary Algorithm 1.** Support vector machine on-board behaviour classification implementation. **Supplementary Algorithm 2.** Artificial neural network on-board behaviour classification implementation. **Supplementary Algorithm 3.** Random forest on-board behaviour classification implementation. **Supplementary Algorithm 4.** Extreme gradient boosting on-board behaviour classification implementation.

## Data Availability

Data of griffon vultures and white storks are available in AcceleRater website: http://accapp.move-ecol-minerva.huji.ac.il/.

## Abbreviations

ACC: Accelerometry
LDA: Linear discriminant analysis
DT: Decision tree
SVM: Support vector machine
RF: Random forest
ANN: Artificial neural network
XGBoost: Extreme gradient boosting
ODBA: Overall dynamic body acceleration
TP: True positive
TN: True negative
FP: False positive
FN: False negative
