# Supplementary material for: An evaluation of machine learning classifiers for next-generation, continuous-ethogram smart trackers
Source: Mov Ecol. 2021 Mar 30;9:15. doi: 10.1186/s40462-021-00245-x (PMC8011142; doi:10.1186/s40462-021-00245-x)
Supplement: Supplementary file 1 — Additional file 1: Supplementary Table 1. Results of parameter tuning of four machine learning methods in five datasets (i.e., for Common crane, Dairy cow, Griffon vulture, Roe deer and Whites stork), with full feature sets and simplified feature sets. Supplementary Table 2. Pseudocodes for feature calculations used for on-board runtime evaluations. Supplementary Algorithm 1. Support vector machine on-board behaviour classification implementation. Supplementary Algorithm 2. Artificial neural network on-board behaviour classification implementation. Supplementary Algorithm 3. Random forest on-board behaviour classification implementation. Supplementary Algorithm 4. Extreme gradient boosting on-board behaviour classification implementation. [file 40462_2021_245_MOESM1_ESM.docx]

Supplementary Table 1. Results of parameter tuning of four machine learning methods in five datasets (i.e., for Common crane, Dairy cow, Griffon vulture, Roe deer and Whites stork), with full feature sets and simplified feature sets. DT: decision tree, SVM: support vector machine, RF: random forest, ANN: artificial neural network.

Supplementary Table 2. Pseudocodes for feature calculations used for on-board runtime evaluations. Under “Note” any dependencies for the calculation of the feature are listed.

| **Index** | **Pseudocode** | **Note** |
| --- | --- | --- |
| 1 | Procedure ***mean()***  array X sum = 0 **for** each value i of X **do**  sum = sum + I **end for** mean = sum / n **return** mean end procedure |  |
| 2 | Procedure ***variance()***  array X sum = 0 **for** each value i of X **do**  sum = sum + pow(i - mean(X), 2) **end for** var = sum / n **return** var end procedure | use 1-mean |
| 3 | Procedure ***standard deviation()***  sd = sqrt(var) **return** sd end procedure | use 2-var |
| 4 | Procedure ***coefficient of variance()***  cv = sd(X) / mean(X) **return** cv end procedure | use 1-mean, 3-sd |
| 5 | Procedure ***skewness()***  array X sum = 0 **for** each value i of X **do**  sum = sum + pow(i - mean(X), 3) **end for** skewness = sum / (n * pow(sd(X), 3)) **return** skewness end procedure | use 1-mean |
| 6 | Procedure ***kurtosis()***  array X sum = 0  **for** each value i of X **do**  sum = sum + pow(i - mean(X), 4) **end for** kurtosis = sum / (n * pow(sd(X), 4)) **return** kurtosis end procedure | use 1-mean |
| 7 | Procedure ***maximum()***  array X max = X[0] **for** each value i of X execpt X[0] **do**  if i > max  max = i  **end if** **end for** **return** max end procedure |  |
| 8 | Procedure ***minimum()***  array X min = X[0] **for** each value i of X execpt X[0] **do**  **if** i < min  min = i   **end if** **end for** **return** min end procedure |  |
| 9 | Procedure ***range()***  range = max(X) - min(X) **return** range end procedure | use 7-max, 8-min |
| 10 | Procedure ***Euclidean norm()***  array X sum = 0 **for** each value i of X **do**  sum = sum + pow(i, 2) **end for** norm = sqrt(sum) **return** norm end procedure |  |
| 11 | Procedure ***covariance()***  array X, Y sum = 0 **for** i from 0 to n -1 **do**  sum = sum + (X[i] - mean(X)) * (Y[i] - mean(Y)) **end for** covxy = sum / n **return** covxy end procedure | use 1-mean |
| 12 | Procedure ***correlation()***  corxy = covxy / (sd(X) * sd(Y)) **return** corxy end procedure | use 3-sd, 11-cov |
| 13 | Procedure ***mean difference()***  array X, Y sum = 0 **for** i from 0 to n - 1 **do**  sum = sum + (X[i] - Y[i])  **end for** meandiffxy = sum / n **return** meandiffxy end procedure |  |
| 14 | Procedure ***standard deviation of difference()***  array X, Y sum = 0 **for** i from 0 to n - 1 **do**  sum = sum + pow(X[i] - Y[i] - meandiffxy, 2) **end for** sddiffxy = sqrt(sum / n) **return** sddiffxy end procedure | use 13-meandiff |
| 15 | Procedure ***variance of static body acceleration()***  array X array static[0:(n - 1)] = 0 p = window length #p is an odd integer na_length = (p - 1) / 2 #center aligned **for** i from (na_length ) to (n - na_length - 1) **do**  sum = 0  **for** j from (i - na_length - 1) to (i + na_length - 1) **do**  sum = sum + X[j]  **end for**  static[i] = sum / p **end for** summean = 0 **for** i from na_length to (n - na_length - 1) **do**  summean = summean + static[i] **end for** meanstatic = sum / (n -p + 1) sumvar = 0 **for** i from na_length to (n - na_length - 1) **do**  sumvar = sumvar + pow(static[i] - meanstatic, 2) **end for** staticvariance = sumvar / (n - p + 1) **return** staticvariance end procedure |  |
| 16 | Procedure ***variance of dynamic body acceleration()***  array X, staticx window = p ##p is an odd integer na_length = (p - 1) / 2 dynamic[0:(n - p)] = 0 **for** i from 0 to (n - p) **do**  dynamic[i] = abs(X[i + na_length] - staticx[i + na_length]) **end for** summean = 0 **for** i from 0 to (n - p) **do**  summean = summean + dynamic[i] **end for** meandynamic = summean / (n - p + 1) sumvar = 0 **for** i from 0 to (n - p) **do**  sumvar = sumvar + pow(dynamic[i] - meandynamic, 2) **end for** dynamicvariance = sumvar / (n - p + 1) **return** dynamicvariance end procedure | use 15-static |
| 17 | Procedure ***mean dynamic body acceleration()***  array dynamicx sum = 0 **for** each i in array dynamicx **do**  sum = sum + i **end for** meandyx = sum / (n - p + 1) **return** meandyx end procedure | use 16-dynamic |
| 18 | Procedure ***overall dynamic body acceleration()***  scalar meandyx, meandyy, meandyz odba = meandyx + meandyy + meandyz **return** odba end procedure | use 17-meandy |
| 19 | Procedure ***maximum dynamic body acceleration()***  array dynamicx maxdba = dynamicx[0] **for** i in array dynamicx except dynamicx[0] **do**  **if** i > max  maxdba = i  **end if** **end for** **return** maxdba end procedure | use 16-dynamic |
| 20 | Procedure ***pitch()***  scalar meanx, meany, meanz pitch = - meanx / sqrt(pow(meany, 2) + pow(meanz, 2)) **return** pitch end procedure | use 1-mean |
| 21 | Procedure ***roll()***  scalar meanx, meany, meanz **if** meanz > 0  sign = 1 **else**   sign = -1 **end if** roll = meany / sign * sqrt(pow(meanz, 2) + 0.001 *pow(meanx, 2)) **return** roll end procedure | use 1-mean |
| 22 | Procedure ***mean difference of continuous points()***  array X array diff[0:(n - 2)] = 0 **for** i from 0 to (n - 2) **do**  diff[i] = abs(X[i + 1] - X[i]) **end for** sum = 0 **for** i from 0 to (n - 2) **do**  sum = sum + diff[i] **end for** meandiff = sum / (n - 1) **return** meandiff end procedure |  |
| 23 | Procedure ***variance of difference of continuous points()***  array diffx, scalar meandiffx sum = 0 **for** each i in array diffx **do**  sum = sum + pow(i - meandiffx, 2) **end for** vardiff = sum / (n - 1) **return** vardiff end procedure | use 22-meandiff |
| 24 | Procedure ***main frequency() and amplitude of main frequency()***  array X array tmp[0:(n - 1)] = 0 tmp = fft(X)  array amplitude[0:(n - 1)] = 0 **for** i from 0 to floor(n/2) - 1 **do**  amplitude[i] = abs(tmp[i]) **end for** ind = 1 mainamp = amplitude[1] **for** i from 1 to floor(n/2) - 1 **do**  **if** amplitude[i] > mainamp  mainamp = amplitude[i]  ind = i  **end if** **end for** mainfreq = ind * fs / n ## fs is sample frequency **return** mainamp, mainfreq end procedure |  |
| 25 | Procedure ***quantiles()***  array X **for** i from 1 to n - 1 **do**  minIndex = I  **for** j from i + 1 to n **do**  if X[j] < X[minIndex]  minIndex = j  **end if**  swap X[i] with x[minIndex]  **end for** **end for** **return** 25%, 50%, 75% quantiles end procedure |  |

Supplementary Algorithm 1: Support vector machine on-board behaviour classification implementation.

Parameters:

**Nc** is number of behaviour classes

**Nf** is number of features used in the SVM classifier

**Ns** is number of support vectors of SVM classifier

**SV** matrix stores values of all support vectors (row-wise)

**nSV** array stores number of support vectors of each behaviour class (therefore, **nSV** has length of **Nc**, and all values summed equals **Ns**)

**coef** matrix stores the corresponding coefficients times the training labels, the dimentsion of this matrix is **Ns** * (**Nc** - 1)

**gamma** is a value used in radial kernel calculation

**newdata** array contains feature values of a new behaviour segment to be classified

**rho** array contains **Nc** negative intercepts

*note:* whether to scale the newdata depends on setting of the trained SVM classifier

///// procedure SVM classifier /////

**function** k_radial(newdata, svm_node, gamma)

{

sum = 0.0

**for** i from 0 to (Nf - 1) **DO**

sum = sum + pow(newdata[i] – svm_node[i], 2)

**end for**

**return** (exp(-gamma*sum))

} /* function for radial kernel calculation */

array kvalue[0:(Ns - 1)] = 0.0

**for** i from 0 to (Ns - 1) **DO**

kvalue[i] = k_radial(newdata, sv[i, ], gamma)

**end for**

array start[0:(Nc - 1)] = 0

**for** i from 1 to (Nc - 1) **DO**

start[i] = start[i - 1] + nSV[i - 1]

**end for**

array vote[0:(Nc - 1)] = 0

p = 0

**for** i from 0 to (Nc - 1) **DO**

**for** j from (i + 1) to (Nc - 1) **DO**

sum = 0.0

si = start[i]

sj = start[j]

ci = nSV[i]

cj = nSV[j]

array coef1 = coef[, j - 1]

array coef2 = coef[, i]

**for** k from 0 to (ci - 1) **DO**

sum = sum + coef1[si + k] * kvalue[si + k]

**end for**

**for** k from 0 to (cj - 1) **DO**

sum = sum + coef2[sj + k] * kvalue[sj + k]

**end for**

sum = sum – rho(p)

**if** sum > 0

vote[i] = vote[i] + 1

**else**

vote[j] = vote[j] + 1

**end if**

p = p + 1

**end for**

**end for**

index = 0

**for** i from 1 to (Nc - 1) **DO**

**if** vote[i] > vote[index]

Index = i

**end if**

**end for**

**return** index /* index is the index of class from this classification procedure */

///// end procedure /////

Supplementary Algorithm 2: Artificial neural network on-board behaviour classification implementation.

Parameters:

**Nf** is number of features

**Nn** is number of nodes in the single layer

**Nc** is number of behaviour classes

**conn** array gives the source unit for the weight (0 = bias unit)

**nconn** array gives the number of first weight connecting to each unit, so the weights connecting to unit i are nconn[i] to nconn[i+1] – 1

**wts** array contains weights for each connection

**newdata** array contains feature values of a new behaviour segment to be classified

**Nunits** = 1 + Nf + Nn + Nc /* 1 represent the bias unit */

///// procedure ANN classifier /////

**function** sigmoid(x)

{

**if** x < -15.0

**return** 0.0

**else if** x > 15.0

**return** 1.0

**else**

**return** 1.0 / (1.0 + exp(-x))

**end if**

} /* function for sigmoid calculation */

array outputs[0:(Nunits - 1)] = 0

outputs[0] = 1.0 /* assign value to the bias unit */

**for** i from 0 to (Nf – 1) **DO**

outputs[i + 1] = newdata[i]

**end for**

**for** j from (Nf + 1) to (Nunits - 1) **DO**

sum = 0.0

**for** i from nconn[j] to (nconn[j + 1] - 1) **DO**

sum = sum + outputs[conn[i]] * wts[i]

**end for**

**if** j <= Nf + Nn

sum = sigmoid(sum) /* values in the nodes of the single layer need sigmoid calculation */

**end if**

outputs[j] = sum

**end for**

index = 1

max = outputs[Nf + Nn + 1]

**for** i from (Nf + Nn + 2) to (Nunits - 1) **DO**

**if** outputs[i] > max

max = outputs[i]

index = i – (Nf + Nn)

**end if**

**end for**

**return** index /* index is the index of class from this classification procedure */

///// end procedure /////

Supplementary Algorithm 3: Random forest on-board behaviour classification implementation.

Parameters:

**Ntree** is number of trees of the random forest classifier

**tree** is a list of all trees

**Nc** is number of behaviour classes

/* The leaf nodes of trees indicate behaviour class. Each tree will give one vote to a behaviour class. Therefore, after traversing all trees, the final behaviour class is the one that has most votes among others. */

///// procedure RF classifier /////

**function** traverse()

{

**while** not leaf node DO

**if** feature value < corresponding threshold

go to left node

**else**

go to right node

**end if**

**end loop**

**return** (behaviour class of the leaf node)

}

array votes[0:(Nc - 1)] = 0

**for** i from 0 to (Ntree - 1) **DO**

class = traverse(tree[i])

votes[class] = votes[class] + 1

**end for**

index = 0

max = votes[0]

**for** i from 1 to (Nc - 1) **DO**

**if** votes[i] > max

max = votes[i]

index = i

**end if**

**end for**

**return** index /* index is the index of class from this classification procedure */

///// end procedure /////

Supplementary Algorithm 4: Extreme gradient boosting on-board behaviour classification implementation.

Parameters:

**Nrounds** is number of iterations of the XGBoost classifier

**tree** is a list of all trees

**Nc** is number of behaviour classes

**Ntree** = Nrounds * Nc is total number of trees

/* The leaf nodes of trees represents raw score for behaviour classes. In each iteration of XGBoost classifier, Nc trees will be trained and each tree works for one behaviour class. The final raw score of one behaviour will be the sum of raw scores from all corresponding trees. */

///// procedure XGBoost classifier /////

**function** traverse()

{

**while** not leaf node **DO**

**if** feature value < corresponding threshold

go to left node

**else**

go to right node

**end if**

**end loop**

**return** (raw score of the leaf node)

}

array scores[0:(Ntree - 1)] = 0.0

**for** i from 0 to (Ntree - 1) **DO**

scores[i] = traverse(tree[i])

**end for**

array finalscores[0:(Nc - 1)] = 0.0

**for** i from 0 to (Nc - 1) **DO**

**for** j from 0 to (Nrounds - 1) **DO**

finalscores[i] = finalscores[i] + scores[i + j * Nc]

**end for**

**end for**

index = 0

max = finalscores[0]

**for** i from 1 to (Nc - 1) **DO**

**if** finalscores[i] > max

max = finalscores[i]

index = i

**end if**

**end for**

**return** index /* index is the index of class from this classification procedure */

///// end procedure /////
